# Case-Control Study on Occupational Exposure to Extremely Low-Frequency Electromagnetic Fields and the Association with Meningioma

**DOI:** 10.1155/2018/5912394

**Published:** 2018-01-03

**Authors:** Michael Carlberg, Tarmo Koppel, Mikko Ahonen, Lennart Hardell

**Affiliations:** ^1^Department of Oncology, Faculty of Medicine and Health, Örebro University, 701 82 Örebro, Sweden; ^2^Department of Labour Environment and Safety, Tallinn University of Technology, SOC353 Ehitajate Tee 5, 19086 Tallinn, Estonia; ^3^Mid Sweden University, 851 70 Sundsvall, Sweden

## Abstract

**Objective:**

Exposure to extremely low-frequency electromagnetic fields (ELF-EMF) was in 2002 classified as a possible human carcinogen, Group 2B, by the International Agency for Research on Cancer at WHO based on an increased risk for childhood leukemia. In case-control studies on brain tumors during 1997–2003 and 2007–2009 we assessed lifetime occupations in addition to exposure to different agents. The INTEROCC ELF-EMF Job-Exposure Matrix was used for associating occupations with ELF-EMF exposure (*μ*T) with meningioma. Cumulative exposure (*μ*T-years), average exposure (*μ*T), and maximum exposed job (*μ*T) were calculated.

**Results:**

No increased risk for meningioma was found in any category. For cumulative exposure in the highest exposure category 8.52+ *μ*T years odds ratio (OR) = 0.9, 95% confidence interval (CI) = 0.7–1.2, and *p* linear trend = 0.45 were calculated. No statistically significant risks were found in different time windows.

**Conclusion:**

In conclusion occupational ELF-EMF was not associated with an increased risk for meningioma.

## 1. Introduction

Meningioma is an encapsulated, well-demarked, and rarely malignant tumor. It is the most common benign brain tumor that accounts for about 30% of intracranial neoplasms. It develops from the pia and arachnoid membranes that cover CNS. It is slow growing and gives neurological symptoms by compression of adjacent structures. Most common symptoms are headaches and seizures. The incidence is about two times higher in women than in men and meningioma develops mostly among middle aged and older persons [1].

One established risk factor for meningioma is ionizing radiation with usually decades of tumor induction period [2]. Due to the female predominance sex hormones have been suggested to be of importance, although not fully explaining the gender difference [3].

In recent years radiofrequency electromagnetic fields (RF-EMF) have been evaluated as risk factors for brain tumors. In May 2011 the International Agency for Research on Cancer (IARC) evaluated the carcinogenic potential from RF-EMF and the expert group classified RF-EMF in the frequency range 30 kHz–300 GHz as “possibly carcinogenic to humans,” Group 2B [4, 5], based on an increased risk for glioma and acoustic neuroma in human case-control studies. Over the years the evidence has strengthened for an increased risk for these tumor types whereas the results for meningioma are less clear [6–12]. The same research groups as for glioma included also meningioma in their case-control studies with a separate publication on meningioma by Carlberg and Hardell [10].

Extremely low-frequency- (ELF-) EMF was in 2002 classified by IARC as “possibly carcinogenic to humans,” Group 2B, based on an increased risk for childhood leukemia [13]. The association was further supported in a pooled analysis yielding about twofold increased risk for childhood leukemia at exposure level above 0.3–0.4 *μ*T [14].

A review showed an increased risk for unspecified brain tumors in the electronic/electric industry with potential exposure to ELF-EMF [15]. In an occupational case-control study on exposure to ELF-EMF no statistically significant increased risk was found for glioma (*n* = 489) or meningioma (*n* = 197) [16]. Another case-control study on occupational ELF-EMF exposure showed for glioma (*n* = 105), odds ratio (OR) = 1.20, 95% confidence interval (CI) = 0.66–2.17, and for meningioma (*n* = 67) OR = 3.02, 95% CI = 1.10–8.25 [17]. No statistically significant association between ELF-EMF exposure and brain tumors was seen in a meta-analysis including 12 studies yielding OR = 1.10, 95% CI = 0.96–1.26 [18].

The INTEROCC study included 1,939 glioma cases, 1,822 meningioma cases, and 5,404 population controls. Occupational ELF-EMF exposure was analysed using a job-exposure matrix (JEM). No association between lifetime cumulative ELF-EMF exposure was found for glioma or meningioma [19]. However, a positive association between cumulative ELF-EMF 1 to 4 years before the diagnosis/reference date was seen for glioma indicating a promoter effect. In the same time window only a weak association was found for meningioma.

In our case-control studies on brain tumors during 1997–2003 and 2007–2009 [10, 11] lifetime occupations were assessed. An ELF-EMF Job-Exposure Matrix was used for associating occupations with ELF exposure (*μ*T). We analysed data on glioma by calculating cumulative exposure (*μ*T-years), average exposure (*μ*T), and maximum exposed job (*μ*T). Cumulative exposure gave for astrocytoma grade IV (glioblastoma multiforme) in the time window 1–14 years OR = 1.9, 95% CI = 1.4–2.6, *p*, linear trend < 0.001, and in the time window 15+ years OR = 0.9, 95% CI = 0.6–1.3, *p*, linear trend = 0.44 in the highest exposure categories 2.75+ and 6.59+ *μ*T years, respectively. An increased risk in late stage (promotion/progression) of astrocytoma grade IV for occupational ELF-EMF exposure was found [20]. No statistically significant increased risk was found for other types of glioma.

Alternating electric current is the source of ELF-EMFs. Most commonly the exposure to ELF-EMFs is due to appliances operating on 50 Hz mains power (60 Hz in Americas and in some parts of Asia). The current is the main determinant of the exposure to the ELF magnetic fields; the more the electrical power is used, the stronger the magnetic field is. Next to the electrical appliances, high exposure to the ELF-EMFs may be encountered also where electrical power is generated, produced, and distributed via power lines, transformers, and so on.

Electrical motors and other devices incorporating electromagnets are another typical source of high ELF magnetic field. In coils, the number of turns of the wire determines the amplitude of the magnetic field. Therefore occupations involving powerful electrical devices are usually accompanied by strong ELF-EMFs. Such professions include electrical transport operators, but also sewing-machine workers and any other profession involved with high power electrical engines placed in close proximity to the worker. The designation of the profession might not always mean that the worker is exposed to strong magnetic fields; in some companies the ELF-EMF source may be positioned further away from the worker, hence alleviating him/her from the high exposure. Powerful electrical motors and other strong magnetic field sources are also found in many industrial settings; the operator's and other workers' exposure is also determined by the distance to the magnetic field source. For example, workers operating hand-held electrical power tools are exposed to strong magnetic fields, whereas others further away from even stronger sources could be exposed to moderate magnetic field levels. Therefore the design of the work machinery and the layout of the working areas has a crucial role in determining the exposure level.

Among highest ELF exposed occupations are, for example, welders, ore, and metal furnace operators, metal melters, casters, and rolling-mill operators. These workers operate machinery that requires a lot of energy, in the form of electrical power. The supply lines and the operating elements, for example, heating elements, pass through high electrical currents, which in turn generate strong magnetic fields.

Also professions dealing with electrical supply systems may become close to strong magnetic field sources, such as electric power linemen, electric power production plant workers, and power distribution workers.

Our case-control studies had detailed occupational history including job titles, branch of different occupations, and years for the specific jobs. Thus it was possible to calculate ELF-EMF job exposure for cases and controls using a job-exposure matrix (JEM).

## 2. Materials and Methods

Similar methods were used in all of our studies. Detailed information on materials and methods has been published previously [8–10]. In short, 6 administrative regions with oncology centres covering Sweden registered new cancer cases. For 1997–2003, cases and controls covered central Sweden [8], whereas the 2007–2009 study included the whole country [9]. The oncology centres reported new cases with histopathologically verified brain tumor, either benign or malignant, to us during these periods, although the actual reporting interval varied for centre to centre. Both men and women were included aged 20–80 years (1997–2003) and 18–75 years (2007–2009) at the time of diagnosis. Only living cases were included after asking the responsible physician for permission before inclusion in the study. Tumor localisation in the brain was based on reports to the cancer registries and medical records, which were obtained after informed consent from the patients.

Controls were ascertained from the Swedish Population Registry. The registry is continuously updated, so that each person could be traced by a unique ID number. It also records the address to each person. For each case, one control subject of the same gender and in the same 5-year group was drawn at random from the Population Registry. They were assigned the same year for cut-off of all exposure as the year of diagnosis of the respective case. All these controls were used in the analysis of risk of meningioma.

Exposure was assessed using a mailed questionnaire sent to each person. The questionnaire contained a number of questions relating to the overall working history, exposure to different chemicals and other agents, smoking habits, X-ray investigations of the head and neck, and heredity traits for cancer. Regarding use of a mobile phone and cordless phone, time period, average daily use (min per day), use of hands free device, and external antenna in a car were also asked for to account for combined influence of RF and ELF radiation. The ear mostly used during phone calls, or equally both, was also noted. Use of the wireless phone was referred to as ipsilateral (>50% of the time) or contralateral (<50% of the time) in relation to tumor side. The same method was also applied for the control group; the subjects were assigned the same “tumor” side as the respective case to the matched control.

When questionnaire answers were unclear, they were resolved by phone using trained interviewers. Thereby, a written protocol was used for clarification of each question. The interviewer supplemented the whole questionnaire during the phone call. Each questionnaire had received a unique ID number that did not disclose whether it was a case or a control; that is, the interviewer was unaware of the status during further data processing. All information was coded and entered into a database. Case or control status was not disclosed until statistical analyses were undertaken.

In this study we included meningioma cases. As comparison group all controls were used. This was possible since we adjusted for potential confounding factors such as year of diagnosis (each control had the same year of “diagnosis” as the respective case), age at diagnosis, gender, and socioeconomic index (SEI).

The questions regarding occupations included job title, branch, and first and last year for each job in the work history of each participant. The INTEROCC ELF Magnetic Field Job-Exposure Matrix (ELFJEM) was used for associating occupations with ELF exposure (*μ*T) [19]. The JEM used International Standard Classification of Occupations 1988 (ISCO88) 4-digit codes for most jobs included; ISCO68 5-digit codes were used for more specific electrical jobs. The online version of the JEM is available at http://www.crealradiation.com/index.php/en/databases?id=55. Job titles were coded using the Nordisk Yrkesklassificering (NYK 85; five digit codes) system and their validity was checked before they were translated to the International Standard Classification of Occupations 1988 (ISCO88; four digit codes) using a coding key provided by Dr. Bihagen at Stockholm University [21]. For translation to the 1968 ISCO version for specific jobs (ISCO68; five digit codes) we compared with the NYK 85 system manually and selected the most proper codes to be translated. Job exposure the year before diagnosis was excluded [20].

Of all cases with a benign tumor 2,068 participated (88%) and most had a meningioma (*n* = 1,625; 79%). Of these 33 were excluded since they had no job codes registered. Thus, in this analysis 1,592 meningioma cases were included. Of all controls 3,530 participated (87%); 45 of them were excluded since they had no job code registered leaving 3,485 included in the analysis.

## 3. Statistical Methods

The analysis was done using StataSE 12.1 (Stata/SE 12.1 for Windows; StataCorp., College Station TX). Odds ratios (OR) and 95% confidence intervals (CI) were calculated using unconditional logistic regression including the whole control sample (i.e., matched to both malignant and benign cases) to increase the power.

Cumulative exposure (*μ*T-years), average exposure (*μ*T), and maximum exposed job (*μ*T) were calculated for the included cases and controls for lifetime work history and in time windows 1–14 and 15+ years before diagnosis. Cut points at the 25th, 50th, 75th, and 90th percentile for controls were used to categorize the exposure variables with the lowest category (<25th percentile) as reference group (OR = 1.0). Tests for linear trends were performed using the Wald test with the median of each category included as an ordinal variable in the analyses. In all analyses adjustment was made for the matching variables gender, age (as a continuous variable), and year of diagnosis and also for socioeconomic index (SEI) divided into three categories (blue-collar worker, white-collar worker, and self-employed).

Restricted cubic splines were used to show the relationship between cumulative exposure to ELF-EMF (*μ*T-years) in time windows and meningioma. Four knots were used at the 5th, 35th, 65th, and 95th percentiles, as suggested by Harrell Jr. [22].

## 4. Results

The mean age of the cases was 57 years (median 57, range 20–80) and of the controls 54 years (median 56, range 20–80). Of the meningioma cases there were 426 men and 1,166 women, versus 1,472 men and 2,013 women in the controls. The mean number of jobs for cases was 2.7 (median = 2, min = 1, max = 11) and for controls 2.7 (median = 2, min = 1, max = 12).

Table 1 displays cumulative exposure in *μ*T-years, average exposure in *μ*T, and maximum exposure job (*μ*T). No statistically significant increased or decreased risk was found for any of the studied variables and we found no statistically significant linear trend for increasing exposure. Cumulative exposure in the highest exposure category, 8.52+ *μ*T-years, gave OR = 0.9, 95% CI = 0.7–1.2, *p*, linear trend = 0.45.

**Figure 1:**
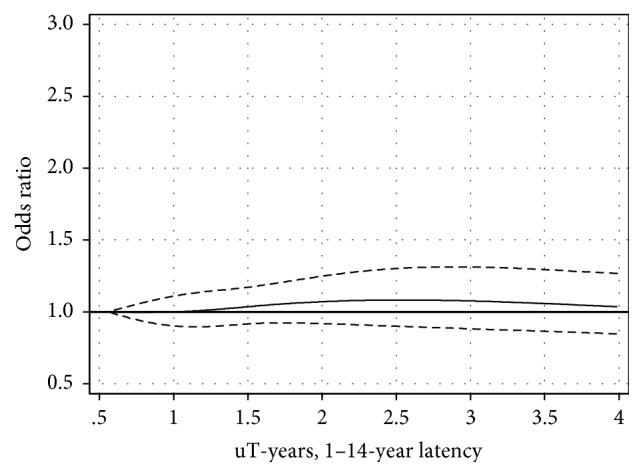
Restricted cubic spline plot of the relationship between cumulative exposure to ELF-EMF in *μ*T-years and meningioma in the 1–14-year latency group. The solid line shows the OR estimate and the broken lines represent the 95% CI. Adjustment for age at diagnosis, gender, SEI-code, and year of diagnosis was made.

**Figure 2:**
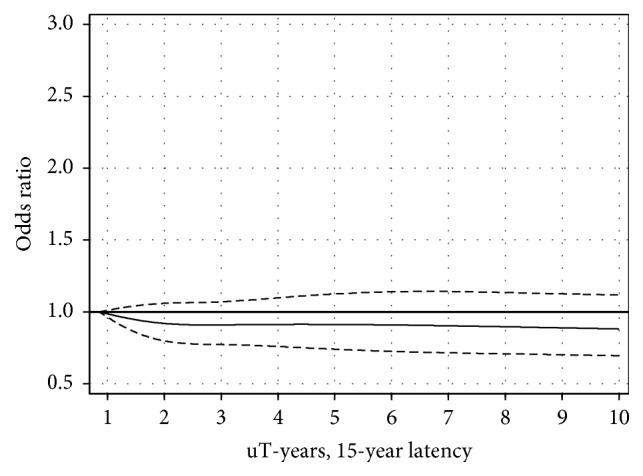
Restricted cubic spline plot of the relationship between cumulative exposure to ELF-EMF in *μ*T-years and meningioma in the 15+ years' latency group. The solid line shows the OR estimate and the broken lines represent the 95% CI. Adjustment for age at diagnosis, gender, SEI-code, and year of diagnosis was made.

Cumulative exposure in different time windows before diagnosis is shown in Table 2 and Figures 1 and 2. No statistically significant risks or linear trends were found. Cumulative exposure in the highest exposure group 2.75+ *μ*T-years yielded OR = 1.0, 95% CI = 0.8–1.3 (*p*, linear trend = 0.71) in the latency group 1–14 years; see Figure 1. For longer latency time, 15+ years, OR = 0.8, and 95% CI = 0.6–1.1 were calculated in the highest exposure group 6.59+ *μ*T-years (*p*, linear trend = 0.28); see Figure 2.

In a separate analysis we grouped latency in 1–4, 5–9 and 10+ years. In the highest exposure category 0.69+ *μ*T-years we calculated OR = 0.9, 95% CI = 0.7–1.2 for tumor induction period 1–4 years, exposure 0.92+ *μ*T-years OR = 1.0, 95% CI = 0.8–1.3 for latency 5–9 years, and exposure 7.28+ *μ*T-years gave OR = 1.0, 95% CI = 0.7–1.3 for latency 10+ years. There was no statistically significant trend (data not in table).

## 5. Discussion

We included in our case-control studies all brain tumors with a diagnosis based on histopathology. The response rate was high among both cases and controls. The two largest groups of cases were glioma and meningioma. Results for occupational ELF-EMF exposure and glioma have been published previously [20].

INTEROCC found a weak association between cumulative occupational ELF-EMF exposure in the time window 1–4 years and meningioma [19]. Thus, the highest exposure category 0.80+ *μ*T years yielded OR = 1.23, 95% CI = 0.97–1.57 with a statistically significant trend (*p* = 0.02). In contrast, INTEROCC reported an increased risk for glioma in late stage carcinogenesis, especially for all glioma, whereas we found increased risk for in late stage carcinogenesis for astrocytoma grade IV (glioblastoma multiforme) only [20].

Similarly, as in the Interphone study on brain tumor risk in relation to mobile telephone use [6], our results on use of wireless phones and brain tumor risk were based on case-control studies. We used a structured questionnaire but here with certain differences regarding the Interphone study, such as that we used postal questionnaires sent to cases and controls supplemented over the phone instead of personal interviews, even bedside interviews of cases as performed in Interphone. Furthermore we assessed in addition to mobile phones also use of cordless phones (DECT); the latter use was not assessed by Interphone. Detailed comparison of the studies may be found elsewhere [23].

Our results were based on a large sample of cases and controls representing a high percentage of participation. Thus, selection bias would not influence the results. Regarding reporting of occupations recall bias is unlikely to be a concern since people tend to give an adequate statement of their previous jobs. If unclear the supplementary phone interview made clarifications using a structured protocol. The different results for astrocytoma grade IV and meningioma in the same study strengthen further the validity of the results for both tumor groups.

A JEM is calculated on samples of same type of occupations; no individual measurements were carried out for the cases/controls in the present study. However, work stations and work assignments may vary within each category of occupations. Thus, exposure to ELF-EMF on an individual basis may not have been correct. However, such differences are likely to be nondifferential. An influence on the reported job is unlikely since mostly ELF-EMF exposure would be unknown for the worker.

Animal studies on ELF-EMF exposure alone have been inconclusive. Long-term ELF-EMF exposure was a risk factor for chronic myeloid leukemia in female mice [24]. Rat studies showed that exposure to ELF-EMF enhanced the carcinogenic effect of *γ* radiation [25] and that life-span exposure to ELF-EMF and formaldehyde induced statistically significant carcinogenic effect [26].

In a recent study ELF-EMF promoted a more malignant phenotype in neuroblastoma cells [27]. ELF-EMF induced a proliferative and survival advantage by activating key redox-responsive antioxidative and detoxification cytoprotective pathways associated with a more aggressive behaviour of neuroblastoma cells. Thus, these results support our previous epidemiological findings of late stage increased risk for glioblastoma multiforme from occupational ELF-EMF exposure [20].

## 6. Conclusion

In conclusion we found no association between occupational ELF-EMF exposure and meningioma.

## Figures and Tables

**Table 1:** Odds ratio (OR) and 95% confidence interval (CI) for meningioma (*n* = 1,592) for occupational exposure to ELF-EMF. Population based controls (*n* = 3,485) were used. Subjects with no coded occupation were excluded, 33 meningioma cases and 45 controls. Unconditional logistic regression, adjusted for age at diagnosis, gender, socioeconomic index (SEI), and year of diagnosis. Exposure the year before diagnosis was excluded (“1-year lag”).

Exposure metric	Meningioma (*n* = 1,592)		
	Ca/Co	OR	95% CI
Cumulative exposure (*µ*T-years)			
<2.33	366/870	1.0	-
2.33–<3.79	451/872	1.1	0.9–1.3
3.79–<5.55	405/869	0.9	0.8–1.1
5.55–<8.52	241/525	1.0	0.8–1.3
8.52+	129/349	0.9	0.7–1.2
*p, linear trend*			0.45
Average exposure (*µ*T)			
<0.11	388/830	1.0	-
0.11–<0.13	441/912	1.0	0.9–1.2
0.13–<0.18	386/871	1.0	0.9–1.2
0.18–<0.27	260/523	1.3	1.04–1.5
0.27+	117/349	0.9	0.7–1.2
*p, linear trend*			0.85
Maximum exposed job (*µ*T)			
<0.13	426/823	1.0	-
0.13–<0.16	381/812	0.9	0.7–1.04
0.16–<0.24	422/968	0.9	0.8–1.1
0.24–<0.60	245/532	1.1	0.9–1.3
0.60+	118/350	0.8	0.6–1.01
*p, linear trend*			0.18

Cut points at 25th, 50th, 75th, and 90th percentile for controls.

**Table 2:** Odds ratio (OR) and 95% confidence interval (CI) for meningioma (*n* = 1,592) for occupational exposure to ELF-EMF in time windows, 1–14 and 15+ years before diagnosis. Unconditional logistic regression, adjusted for age at diagnosis, gender, socioeconomic index (SEI), and year of diagnosis. Exposure the year before diagnosis was excluded (“1-year lag”).

Cumulative exposure (*µ*T-years)	Meningioma (*n* = 1,592)		
	Ca/Co	OR	95% CI
1–14 years			
<0.91	368/770	1.0	-
0.91–<1.42	391/872	0.9	0.8–1.1
1.42–<1.82	354/778	1.0	0.8–1.2
1.82–<2.75	230/537	1.0	0.8–1.3
2.75+	125/329	1.0	0.8–1.3
*p, linear trend*			0.71
15+ years			
<1.44	418/782	1.0	-
1.44–<2.55	399/777	0.9	0.8–1.1
2.55–<4.17	354/787	0.8	0.6–0.95
4.17–<6.59	220/471	0.9	0.7–1.1
6.59+	120/313	0.8	0.6–1.1
*p, linear trend*			0.28

Cut points at 25th, 50th, 75th, and 90th percentile for controls in each time window.

## References

[1] Cea-Soriano L., Wallander M.-A., Garca Rodrguez L. A. (2012). Epidemiology of meningioma in the United Kingdom. *Neuroepidemiology*.

[2] Braganza M. Z., Kitahara C. M., Berrington De González A., Inskip P. D., Johnson K. J., Rajaraman P. (2012). Ionizing radiation and the risk of brain and central nervous system tumors: A systematic review. *Neuro-Oncology*.

[3] Korhonen K., Salminen T., Raitanen J., Auvinen A., Isola J., Haapasalo H. (2006). Female predominance in meningiomas can not be explained by differences in progesterone, estrogen, or androgen receptor expression. *Journal of Neuro-Oncology*.

[4] Baan R., Grosse Y., Lauby-Secretan B. (2011). Carcinogenicity of radiofrequency electromagnetic fields. *The Lancet Oncology*.

[5] IARC Monographs on the Evaluation of Carcinogenic Risks to Humans *Non-Ionizing Radiation, Part 2: Radiofrequency Electromagnetic Fields*.

[6] Interphone Study Group (2010). Brain tumour risk in relation to mobile telephone use: results of the INTERPHONE international case-control study. *International Journal of Epidemiology*.

[7] Coureau G., Bouvier G., Lebailly P. (2014). Mobile phone use and brain tumours in the CERENAT case-control study. *Occupational and Environmental Medicine*.

[8] Hardell L., Carlberg M., Mild K. H. (2006). Pooled analysis of two case-control studies on the use of cellular and cordless telephones and the risk of benign brain tumours diagnosed during 1997–2003. *International Journal of Oncology*.

[9] Carlberg M., Söderqvist F., Hansson Mild K., Hardell L. (2013). Meningioma patients diagnosed 2007-2009 and the association with use of mobile and cordless phones: A case-control study. *Environmental Health: A Global Access Science Source*.

[10] Carlberg M., Hardell L. (2015). Pooled analysis of Swedish case-control studies during 1997-2003 and 2007-2009 on meningioma risk associated with the use of mobile and cordless phones. *Oncology Reports*.

[11] Hardell L., Carlberg M. (2015). Mobile phone and cordless phone use and the risk for glioma—analysis of pooled case-control studies in Sweden, 1997–2003 and 2007–2009. *Pathophysiology*.

[12] Carlberg M., Hardell L. (2017). Evaluation of mobile phone and cordless phone use and glioma risk using the Bradford Hill viewpoints from 1965 on association or causation. *BioMed Research International*.

[13] IARC Monographs on the Evaluation of Carcinogenic Risks to Humans *Non-Ionizing Radiation, Part 1: Static and Extremely Low-Frequency (ELF) Electric and Magnetic Fields*.

[14] Kheifets L., Ahlbom A., Crespi C. M. (2010). Pooled analysis of recent studies on magnetic fields and childhood leukaemia. *British Journal of Cancer*.

[15] Hardell L., Holmberg B., Malker H., Paulsson L.-E. (1995). Exposure to extremely low frequency electromagnetic fields and the risk of malignant diseases—an evaluation of epidemiological and experimental findings. *European Journal of Cancer Prevention*.

[16] Coble J. B., Dosemeci M., Stewart P. A. (2009). Occupational exposure to magnetic fields and the risk of brain tumors. *Neuro-Oncology*.

[17] Baldi I., Coureau G., Jaffré A. (2011). Occupational and residential exposure to electromagnetic fields and risk of brain tumors in adults: A case-control study in Gironde, France. *International Journal of Cancer*.

[18] Zhang Y., Lai J., Ruan G., Chen C., Wang D. W. (2016). Meta-analysis of extremely low frequency electromagnetic fields and cancer risk: A pooled analysis of epidemiologic studies. *Environment International*.

[19] Turner M. C., Benke G., Bowman J. D. (2014). Occupational exposure to extremely low-frequency magnetic fields and brain tumor risks in the INTEROCC study. *Cancer Epidemiology, Biomarkers & Prevention*.

[20] Carlberg M., Koppel T., Ahonen M., Hardell L. (2017). Case-control study on occupational exposure to extremely low-frequency electromagnetic fields and glioma risk. *American Journal of Industrial Medicine*.

[21] Bihagen E. (2007). Nya möjligheter för stratifieringsforskning i Sverige: Internationella yrkesklassificeringar och stratifieringsmått över tid. *Sociologisk Forskning*.

[22] Harrell F. E. (2001). *Regression Modeling Strategies. With Application to Linear Models, Logistic Regression and Survival Analysis*.

[23] Hardell L., Carlberg M., Mild K. H. (2008). Methodological aspects of epidemiological studies on the use of mobile phones and their association with brain tumors. *Open Environmental Sciences Journal*.

[24] Qi G., Zuo X., Zhou L. (2015). Effects of extremely low-frequency electromagnetic fields (ELF-EMF) exposure on B6C3F1 mice. *Environmental Health and Preventive Medicine*.

[25] Soffritti M., Tibaldi E., Padovani M. (2016). Life-span exposure to sinusoidal-50 Hz magnetic field and acute low-dose *γ* radiation induce carcinogenic effects in Sprague-Dawley rats. *International Journal of Radiation Biology*.

[26] Soffritti M., Tibaldi E., Padovani M. (2016). Synergism between sinusoidal-50 Hz magnetic field and formaldehyde in triggering carcinogenic effects in male Sprague-Dawley rats. *American Journal of Industrial Medicine*.

[27] Falone S., Santini S., Cordone V. (2017). Power frequency magnetic field promotes a more malignant phenotype in neuroblastoma cells via redox-related mechanisms. *Scientific Reports*.

